# Effects of kappa-opioid receptor stimulation on addiction-related behavior in female rats: differences along the rostro-caudal axis of the nucleus accumbens shell

**DOI:** 10.1007/s00213-025-06931-7

**Published:** 2025-10-23

**Authors:** Annie Hawks, Mary B. Spodnick, Malcolm C. Jennings, Christina M. Nelson, Megan Peng, Gavin Vaughan, Brody A. Carpenter, Chan N. Chung, Anushree N. Karkhanis, Jessica R. Barson

**Affiliations:** 1 Barson Lab, Department of Neurobiology and Anatomy, Drexel University College of Medicine, Philadelphia, PA 19129, USA; 2 Karkhanis Lab, Department of Psychology, Binghamton University - SUNY, Binghamton, NY 13902, USA

**Keywords:** Caudal NAc, Ethanol, Intermittent access, Light-dark box, Long-Evans, Progressive ratio, Rostral NAc, Self-administration, Sucrose, U50,488

## Abstract

**Rationale:**

Affective behavioral effects of kappa-opioid receptor (KOR) stimulation have been found to vary between the rostral and caudal nucleus accumbens (NAc) shell, but this has so far only been demonstrated in males.

**Objectives:**

To examine the effects of KOR activation in subregions of the NAc shell on affective and motivated behavior in females.

**Methods:**

In one group of Long-Evans rats, females were given access to 20% ethanol in an intermittent-access procedure and then injected within-subject with the selective KOR agonist, U50,488, or vehicle in the rostral or caudal NAc shell, prior to being tested in a light-dark box or having their ethanol drinking monitored. In a second group of Long-Evans rats, females and males were trained to self-administer 10% sucrose and then injected within-subject with U50,488 or vehicle in the rostral or caudal NAc shell, prior to being tested in a progressive ratio (PR) schedule of reinforcement.

**Results:**

Injection of U50,488 into the caudal but not rostral NAc shell reduced the number of entries into the light chamber of a light-dark box, while injection of U50,488 into the rostral but not caudal NAc shell reduced ethanol drinking. Injection of U50,488 into neither the rostral nor the caudal NAc shell had any effect on any measure of sucrose intake.

**Conclusions:**

The present findings confirm and extend previous findings regarding the effects of KOR activation in subregions of the NAc shell on affective and motivated behavior. These results support a continued focus on the KOR as a possible pharmacotherapeutic target.

## Introduction

Activation of the kappa-opioid receptor (KOR) has canonically been associated with negative affective states (Jacobson et al. 2020a; Land et al. 2008) and has been implicated in conditions that include anxiety disorders (Khan et al. 2022; Limoges et al. 2022), alcohol use disorders (Ozkan-Kotiloglu et al. 2023; Park et al. 2020), and eating disorders (Engin 2024; Hasan and Hasan 2011; Karkhanis et al. 2017). Limited research, however, has demonstrated that KOR activation in some cases can be anxiolytic and lead to increases in reward (Chartoff et al. 2016; Kudryavtseva et al. 2006; Privette and Terrian 1995; Wright et al. 2018), as well as mixed effects on ethanol drinking (Kudryavtseva et al. 2006; Lindholm et al. 2001; Zhou et al. 2020) and eating (Yonemochi et al. 2024). This suggests that KOR activation could have different effects on behavior through different brain regions.

One region where we and others have found opposing effects of KOR activation on affective and motivated behavior is the nucleus accumbens (NAc). Studies have identified dense gene expression and binding of the KOR in the NAc, particularly its shell region, in both the mouse and rat (Chen et al. 2020; Mansour et al. 1995; Marinelli et al. 2000). In male rats, Castro and Berridge found that conditioned place preference as well as positive orofacial reactions to sucrose taste infusion were induced by microinjection of the selective KOR agonist, U50,488, into the rostral half of the NAc shell (Castro and Berridge 2014). Conversely, they identified an increase in conditioned place aversion and a suppression of positive orofacial reactions after microinjection of U50,488 into the caudal shell (Castro and Berridge 2014). Similarly, we found in male rats that microinjection of U50,488 into the rostral NAc shell led to more entries into the center of an open field, while microinjection into the caudal NAc shell instead reduced time spent in the light chamber of a light-dark box and reduced ambulatory distance in an open field (Pirino et al. 2020). We also found that microinjection of U50,488 into the rostral NAc shell reduced ethanol drinking in male rats and low-drinking females, whereas there was no such effect with microinjection into the caudal NAc shell (Pirino et al. 2024). Collectively, these results suggest that KOR stimulation in the rostral NAc shell is likely to induce approach or anxiolytic-type behaviors, along with a reduction in ingestive behaviors, whereas KOR stimulation in the caudal NAc shell is likely to induce the opposite behavioral effects. How these effects may differ in female rats, and how these effects on affective behavior may interact with ethanol drinking, remains to be determined.

Sex-related differences have been identified in anxiety-like or approach-avoidance behavior, sucrose drinking, and the KOR system in the NAc. In a light-dark box test, with no effect of the estrous cycle on these behaviors, females have sometimes but not always been found to spend more time in the light chamber (Davis et al. 2008; De Oliveira Sergio et al. 2021; Lorente et al. 2024; Pirino et al. 2022), make more entries into the light chamber (De Oliveira Sergio et al. 2021; Fleming et al. 2019; Kokras et al. 2021; Pirino et al. 2022), and engage in more overall ambulatory activity than males (Davis et al. 2008; Gillette et al. 2017; Pirino et al. 2022; Viviani et al. 2012). A history of ethanol exposure may inhibit these behaviors (Fleming et al. 2019; Pirino et al. 2022). In an intermittent or limited access model of sucrose intake, females have been found to drink more sucrose and engage in more sucrose self-administration than males (Lopez et al. 2023; Pirino et al. 2024). In contrast, females appear to be less sensitive than males to the behavioral effects of U50,488 (Conway et al. 2019; Jacobson et al. 2020b), despite showing no difference in KOR gene expression in the NAc (Conway et al. 2019). A history of ethanol exposure may upregulate these levels (Cuitavi et al. 2024; Niinep et al. 2021; Pirino et al. 2024). Therefore, prior findings of rostro-caudal differences in NAc shell KOR activation on behavior may not necessarily be similar in females.

The purpose of this study was to examine the effects of KOR activation in subregions of the NAc shell on affective and motivated behavior in female rats, for comparison with findings in male rats. In the first experiment, we examined female rats with a history of intermittent-access ethanol drinking, to determine the effects of U50,488 injection in the rostral compared to caudal NAc shell on behavior in a light-dark box and ethanol drinking. Then, in the next experiment, we examined both female and male rats, to determine the effects of U50,488 injection in the rostral compared to caudal NAc shell on sucrose intake and seeking. We hypothesized that, as has been reported with males, females would show opposing effects of rostral and caudal NAc shell KOR stimulation.

## Materials and methods

### Animals and housing

For Experiment 1, adult, female Long-Evans rats (*N* = 32, 7 weeks on arrival at the facility, Charles River Laboratories International, Inc., Malvern, PA, USA) were individually housed in an AAALAC-accredited facility, on a 12-hour reversed light/dark cycle (lights off at 0900 h). They were given one week to acclimate to the facility. Animals received *ad libitum* chow (Laboratory Rodent Diet 5001, Lab Diet, St. Louis, MO, USA) and water throughout the study. Experiments were approved by the Institutional Animal Care and Use Committee of Drexel University College of Medicine and followed the NIH Guide for the Care and Use of Laboratory Animals. For Experiment 2, adult male and female Long-Evans rats (*N* = 14 (male) and 12 (female); 8 weeks on arrival at the facility, Envigo, Indianapolis, IN, USA) were housed in an AAALAC-accredited facility, on a 12-hour reversed light/dark cycle (lights off at 0300 h). Animals received *ad libitum* chow (5L0D – PicoLab Laboratory Rodent Diet, Lab Diet) and water throughout the study. For this experiment, rats were pair-housed during the one week of acclimation to the facility, and then subsequently individually housed. Experiments were approved by the Institutional Animal Care and Use Committee of Binghamton University and followed the NIH Guide for the Care and Use of Laboratory Animals. Estrous cycle was not assessed, to minimize stress and reduce the chances of pseudo-pregnancy (Lovick and Zangrossi 2021; Singletary et al. 2005).

## Detailed methods

### Experiment 1 – Effects of KOR stimulation on behavior in a light-dark box and ethanol drinking

#### Experimental protocol

**Fig. 1:**
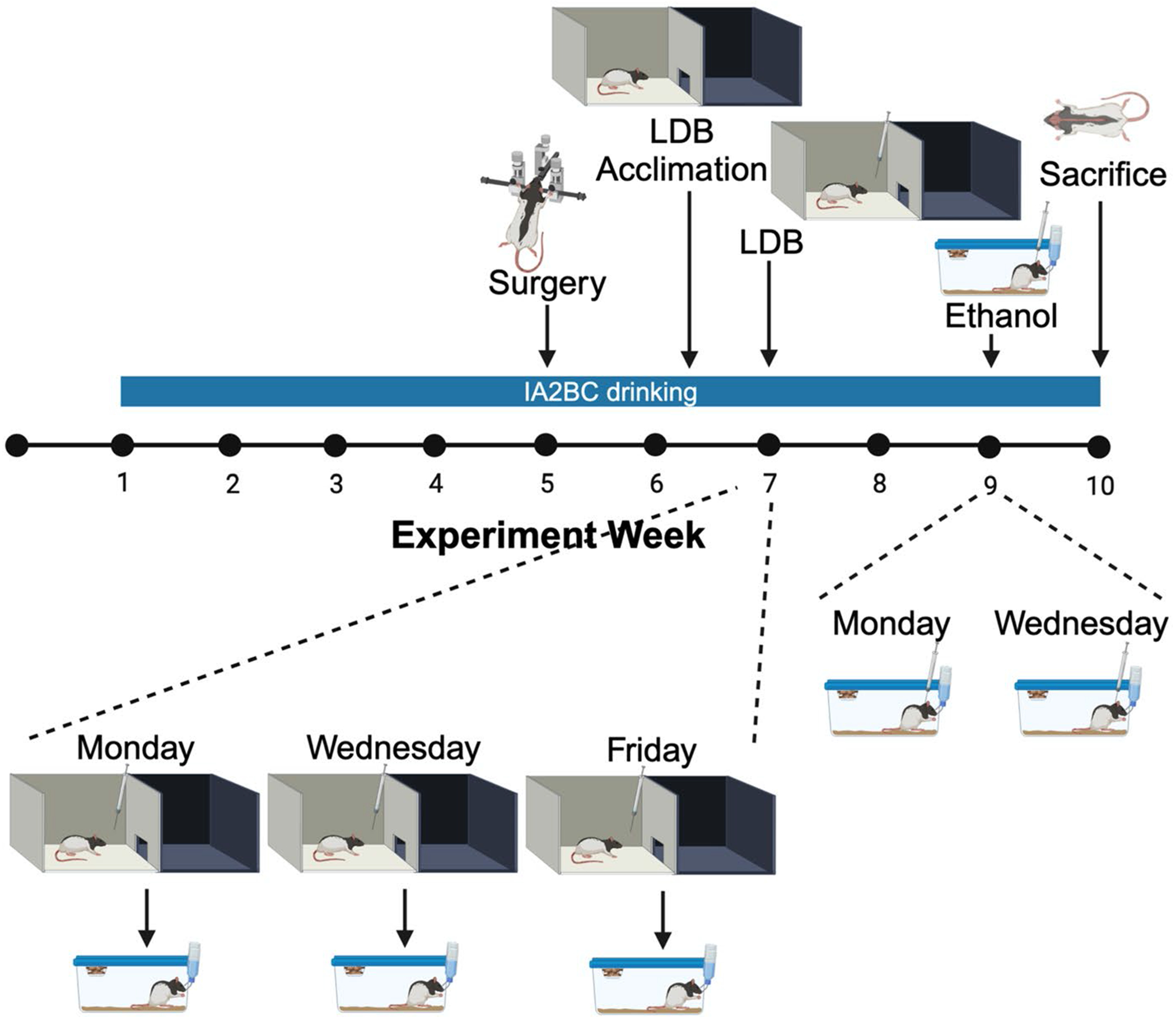
Experimental timeline for Experiment 1. Female rats were given access to 20% v/v ethanol in an intermittent-access two-bottle-choice procedure and bilaterally cannulated in the rostral or caudal nucleus accumbens shell during the 5th week of drinking. At the end of the 6th week of drinking, they were acclimated to the light-dark box. During the 7th week of drinking, they were injected with two doses of U50,488 counterbalanced against saline vehicle across three ethanol access days for testing in the light-dark box. After one-to-two weeks for recovery, they were again injected with U50,488 counterbalanced against saline vehicle across two ethanol access days, for testing of ethanol intake. In the final week, they were sacrificed for histological examination. Created with BioRender.com

See Fig. 1 for a schematic representation of the protocol for Experiment 1. To determine if the differential effects on affective behavior of KOR stimulation in subregions of the NAc shell extend to females after a history of ethanol drinking, female rats (*N* = 32, run in two cohorts of 16 rats each) were given access to 20% v/v ethanol in an intermittent-access two-bottle-choice procedure and bilaterally cannulated during the 5th week of drinking, after ethanol drinking levels were expected to be stable (Pirino et al. 2022), for injections into the rostral or caudal NAc shell. At the end of the 6th week of drinking (Friday or Thursday), they were acclimated to the light-dark box, by being placed in the box for 5 min. The following week, they were injected 10 min prior to testing in the light-dark box with U50,488 (0.8 nmol or 8.0 nmol) counterbalanced against saline vehicle in a within-subject Latin-square design across three ethanol access days. After one (cohort 1)-to-two (cohort 2) weeks for recovery, they were again injected 10 min prior to testing with U50,488 (8.0 nmol) counterbalanced against saline vehicle in a within-subject Latin-square design across two ethanol access days, and subsequent ethanol intake was measured at 30 min, the period during which animals drink ethanol in a binge-like manner (Pirino et al. 2022). Due to poor health, faulty cannulas, or off-target cannulations, data from 11 females were not included in the analysis.

#### Ethanol drinking

Under the intermittent-access two-bottle-choice procedure adapted from Wise (1973) and Simms et al. (2008), rats were given access to unsweetened 20% v/v ethanol (diluted with tap water from 190 Proof ethanol, Deacon Labs, King of Prussia, PA) during three 24-hour-sessions per week in addition to *ad libitum* water and chow as described (Barson et al. 2015; Gargiulo et al. 2021; Pirino et al. 2022). Each Monday, Wednesday, and Friday (or Sunday, Tuesday, and Thursday), one-and-a-half hours after dark onset, one of their two 16 oz Macrolon bottles of water (Ancare, Bellmore, NY, USA) was replaced with a 9 oz polycarbonate bottle of ethanol (Ancare) and, after 24 h, the ethanol was replaced with the second bottle of water. Relative bottle position was alternated each time to prevent side preference, and all bottles were fitted with non-drip sipper tubes of equal size. Ethanol intake was calculated as: (weight ethanol solution consumed (g) * (density ethanol * 0.20)/rat body weight (kg). Animals were weighed on Tuesdays and Fridays (or Mondays and Thursdays).

#### Stereotaxic surgery

Rats were bilaterally cannulated using published methods (Pirino et al. 2020). Cannulas (made from 21-gauge stainless steel tubing, Small Parts at Amazon.com, Seattle, WA, USA) were implanted perpendicularly, aimed at the rostral or caudal NAc shell (1.9–0.8 mm anterior to Bregma, ± 0.8 mm lateral to midline, 3.5 mm ventral to the level skull) (Paxinos and Watson 2014). Bupivicaine (2 mg/kg s.c., Hospira Worldwide, Lake Forest, IL, USA) was injected into the scalp prior to incision, and buprenorphine hydrocholoride (0.03 mg/kg s.c., Reckitt & Colman Inc, Slough, UK) was administered for post-operative analgesia. To prevent occlusion, stylets (made from 26–gauge stainless steel tubing, Small Parts) were left in the cannulas between injections. Rats were given one week to recover from surgery prior to the start of microinjections, and animals were handled daily during this time, with their stylet removed and replaced to acclimate them to the microinjection procedure.

#### Drugs

The selective KOR agonist (±)-trans-U50,488 hydrochloride (U50,488) was acquired from Tocris (Minneapolis, MN, USA) and dissolved in 0.9% saline (Baxter International Inc., Deerfield, IL, USA) for microinjection at 0.8 nmol and 8.0 nmol per side (Pirino et al. 2020) in a volume of 0.3 μL. These doses have previously been found to alter behavior after injection into the NAc shell of male and female rats (Nealey et al. 2011; Pirino et al. 2020, 2024). They have been demonstrated to be within the range of KOR selectivity (Jenck et al. 1988; Massaly et al. 2019).

#### Microinjections

Rats were injected with freshly prepared U50,488 or saline vehicle 1.5–2 h into the dark cycle, through microinjectors of 26–gauge stainless steel outside and fused-silica tubing inside (74 μm ID, 154 μm OD; Polymicro Technologies, Phoenix, AZ, USA) that extended 4.0 mm beyond the cannulas to reach the NAc shell. A syringe pump (Harvard Apparatus, Holliston, MA, USA) delivered 0.3 μl of solution over 30 s, and the microinjector remained in place for an additional 30–60 s to allow for diffusion. Each side was injected sequentially, such that injection in one hemisphere for a single subject was immediately followed by injection into the other hemisphere. We have previously demonstrated that injections of methylene blue dye or a fluorescent dextran at the volume used (0.3 μl) have a radial spread of approximately 0.5 mm and largely remain restricted within the NAc shell (Barson et al. 2015; Gargiulo et al. 2021). With the NAc shell estimated to span almost 3 mm along the anterior-posterior axis of an adult rat, this suggests that our microinjections were largely restricted within each subregion.

##### Light-dark box testing

Behavioral testing was conducted in a sound- and light-attenuated room (< 5 lx), starting 1.5–2 h into the dark cycle, at a time when animals would normally receive ethanol (24–48 h into abstinence). Rats were brought from the vivarium to the testing room, with their cages fully covered with an opaque drape, and were given 5 min to acclimate to the testing room prior to the start of the experiment. A light/dark insert was placed in an automated activity chamber with an area of 43.2 cm x 43.2 cm and 42 cm high walls (Med Associates, Inc., St. Albans, VT, USA), to create a two-chamber light-dark box. A lamp was placed directly above the light chamber, creating a luminous intensity of approximately 400 lx in that chamber. Animals were placed in the light chamber, facing the dark chamber, and allowed to explore the light-dark box for 5 min. Time spent engaged in horizontal activity (“ambulatory time”), horizontal distance traveled (“ambulatory distance”), time spent in the light chamber (“light time”), and number of entries into the light chamber (“light entries”) were measured via infrared beams (Gargiulo et al. 2021; Pirino et al. 2020, 2022). The first 5-minute session in the light-dark box was used for acclimation to the chamber, as we and others have found that behavior during the first time in the light-dark box is different from behavior during subsequent times (Bouwknecht et al. 2004; Pirino et al. 2022; Rodgers and Shepherd 1993). Subsequent 5-minute sessions in the light-dark box were used for the tests. Rats were given access to their scheduled ethanol following the conclusion of the test, once they were returned to their vivarium room.

#### Histological analysis

Rats were sacrificed at the end of the experiment by rapid decapitation after brief CO2 exposure, and their brains were extracted. Microscopic examination of cresyl violet- (Nissl-) stained 30 μm coronal sections was used to ensure that injections were made into the medial NAc shell and determine the rostro-caudal subregion at which this occurred. Bregma + 3.00 – +1.92 mm was considered rostral and Bregma + 1.80 – +0.84 mm was considered caudal (Paxinos and Watson 2014).

#### Statistical analysis

Given our prior findings of differences in the effects of U50,488 between the rostral and caudal NAc shell on behavior in a light-dark box and ethanol drinking in males (Pirino et al. 2020, 2024), an a priori decision was made to directly test effects of U50,488 in each subregion, rather than compare the effects across subregions. To compare ethanol drinking between groups prior to the start of microinjections, a mixed ANOVA was used, with cannnulation area as the between-subject measure and drinking week as the within-subject measure. To compare behavior between groups during the acclimation to the light-dark box, independent two-tailed *t*-tests were used. To examine the relationship between average weekly ethanol intake prior to testing and behavior during the acclimation to the light-dark box, linear regressions were used. To compare behavior after injections with the different doses of U50,488, a repeated-measures ANOVA was used, with dose as the within-subject measure. Significant main effects were followed up with a Sidak pairwise comparison test. To determine effects of U50,488 on ethanol drinking, paired-sampled two-tailed *t*-tests were used. To examine the relationship between the effect of U50,488 on behavior in the light-dark box and ethanol drinking, the change in each behavior after the high dose of U50,488 was calculated as a percent change using the equation [((measure after high dose of U50,488 – measure after saline)/(measure after saline)) * 100], and linear regressions were used. Sphericity was determined using Mauchly’s test, and a Greenhouse-Geisser correction was used when sphericity was violated. Significance was determined at *p* < 0.05.

### Experiment 2 – Effects of KOR stimulation on lever-pressing for sucrose drinking

#### Experimental protocol

**Fig. 2:**
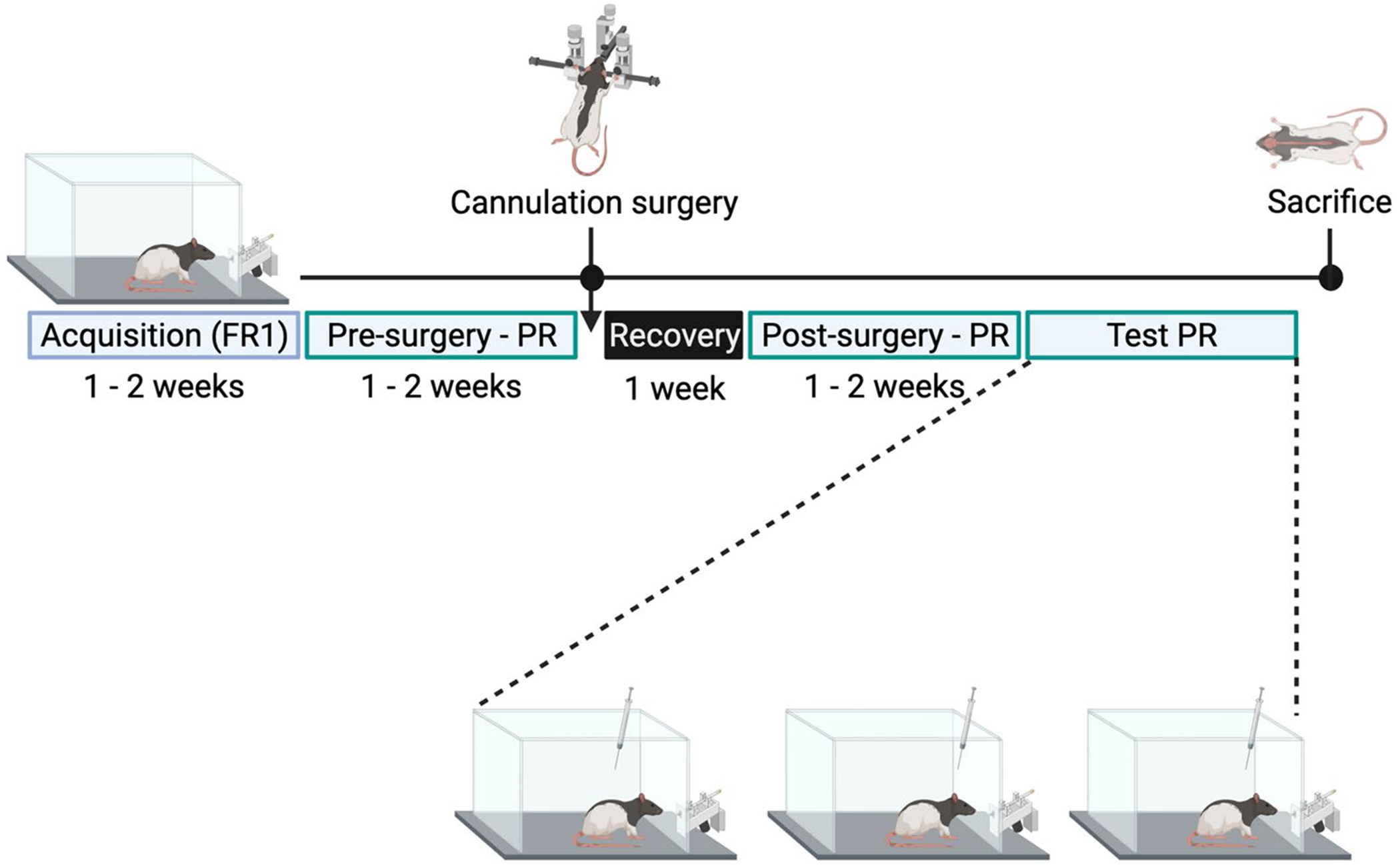
Experimental timeline for Experiment 2. Male and female rats were given access to 10% w/v sucrose on an fixed ratio 1 (FR1) schedule of reinforcement. After rats exhibited successful association between the lever and sucrose reward (3 days of responses with a minimum of 10 active lever presses and/or 200 licks), rats were transitioned to a progressive ratio (PR) schedule of reinforcement. After reaching stable responding (number of rewards earned varied by no more than two over the course of three consecutive days), rats underwent cannula implantation surgery followed by 7 days of recovery. On the 8th day post-surgery, rats were placed back in the operant chambers and given access to self-administer 10% sucrose under the PR schedule of reinforcement. Once stable responding on PR was reestablished, rats were injected with two doses of U50,488 counterbalanced with sterile water vehicle into the rostral or caudal nucleus accumbens shell, before being placed in the operant chamber to self-administer sucrose with PR. At the end of the experiment, rats were sacrificed and brains were harvested to verify cannula placements. Created with BioRender.com

See Fig. 2 for a schematic representation of the protocol for Experiment 2. To determine if the differential effects of KOR stimulation in subregions of the NAc shell on light-dark box behavior and ethanol drinking extend to lever-pressing for sucrose drinking, male and female rats were trained to self-administer 10% w/v sucrose solution first using a fixed ratio 1 (FR1) schedule of reinforcement, and then a progressive ratio (PR) schedule of reinforcement. Microinjection cannulas were implanted bilaterally once PR responding was stable (after at least 7 days). PR responding was reestablished post-surgery. Once stable, rats were injected 15 min prior to testing on PR with U50,488 (0.8 nmol or 8.0 nmol) counterbalanced against sterile water vehicle across days in a within-subject Latin-square design, with at least 48h between the microinjections to allow for full clearance of U50,488 by the animal. Rats were maintained on the PR schedule of reinforcement at regular experiment times in between microinjections.

#### Sucrose self-administration

Rats were trained to self-administer sucrose solution in tap water (10%, w/v) using operant chambers (ENV-016MD, MED Associates, St. Albans, Vermont, USA) containing cue lights, an active and an inactive lever (ENV-122CM), and sipper tubes (ENV-252 M). Chambers were programmed and operated using MED-PC. Self-administration sessions were conducted during the dark cycle Monday through Friday at 0830 (session 1) and 1230 (session 2), with each rat being run at the same time/session in the same chamber. Rats were first trained on a FR1 schedule of reinforcement, in which a single lever press resulted in access to the sipper tube for 30 s. Once rats exhibited behavior indicative of task acquisition (3 days of responses with a minimum of 10 active lever presses and/or 200 licks), they were transitioned to a PR schedule of reinforcement, to assess sucrose seeking. In the PR schedule of reinforcement, the number of lever presses required to obtain access to the sipper tube increased progressively in the following sequence: 1, 2, 4, 6, 9, 12, 15, 20, 25, 32, 40, 50, 62, 77, 95, 118, 145, 178, 219, 268, 328, 402, 492, and 603 (Richardson and Roberts 1996). Each session lasted for one hour, during which breakpoints, final response ratio, and sucrose solution consumed was recorded. The breakpoint was defined as the trial at which the rat stopped pressing the lever for the sucrose solution and the final response ratio was defined as the number of times the lever was pressed to obtain the last reward of the session. Rats were employed on the PR schedule of reinforcement for at least seven days or until stable responding was achieved, whichever came second, before cannulation surgery. Stable responding was defined by less than a difference of two in the number of rewards earned over the course of three consecutive days. Rats were then retrained on PR post-surgical recovery. After the first post-surgery recovery day (Day 8 post-surgery), animals continued to perform the PR task on their regular schedule until stable responding was reestablished. An animal was considered to have reached stable PR responding post-surgery when the number of rewards earned varied by no more than two over the course of three consecutive days.

#### Stereotaxic surgery

After at least seven days of PR training, animals were implanted bilaterally with guide cannula (part nos. 8IC235G16XXC and 8IC235G20XXC, PlasticsOne, Roanoke, VA, USA). The cannulas were aimed at either the rostral (2.1 (male) or 1.8 mm (female) anterior to Bregma, ± 0.8–1 mm lateral to midline, 4.0 mm ventral to the level skull) or caudal NAc shell (1.1 (male) or 0.8 mm (female) anterior to Bregma, ± 0.8–1 mm lateral to midline, 4.0 mm ventral to the level skull). Injector dummies (8IC235DCSPCC, PlasticsOne, Roanoke, VA) were left in the cannulas between injections to prevent occlusion. Animals were allowed one week to recover during which their body weights and responsivity were monitored.

##### Drugs

The selective KOR agonist (±)-trans-U50,488 hydrochloride (U50,488) was acquired from Tocris (Minneapolis, MN, USA) and dissolved in sterile water (NDC 0409-4887-10; Hospira Inc., Lake Forest, IL, USA) for microinjection at 0.8 nmol and 8.0 nmol per side in a volume of 0.3 μL. These doses have previously been found to alter behavior after injection into the NAc shell of male and female rats (Nealey et al. 2011; Pirino et al. 2020, 2024) and have been demonstrated to be within the range of KOR selectivity (Jenck et al. 1988; Massaly et al. 2019).

##### Microinjections

Once stable sucrose responding was achieved, rats were microinjected with 0.3 μL of sterile water or U50,488 (0.8 nmol and 8 nmol) dissolved in sterile water, 5–9 h into the dark cycle. Bilateral injectors (81C235ISPCXC, PlasticsOne, Roanoke, VA) extended 3.0 mm beyond the cannulas, to reach the NAc shell. A syringe pump (Harvard Apparatus, Holliston, MA, USA) delivered 0.3 μl of solution over 30 s, and the microinjector remained in place for an additional 30–60 s to allow for diffusion.

#### Cannula placement confirmation

Rats were sacrificed at the end of the experiment by rapid decapitation after isoflurane and microinjected with 0.3 μL of 1% methylene blue dye (M9140, MilliporeSigma, St. Louis, MO, USA). Brains were extracted and sliced coronally in 300 μm slices using a vibrating tissue slicer (VT1200 S, Leica BioSystems, Buffalo Grove, IL, USA). Cannula placement was confirmed by observing the injected dye and comparing the coronal sections to those represented in a stereotaxic atlas. Bregma + 3.00 – +1.92 mm was considered rostral and Bregma + 1.80 – +0.84 mm was considered caudal (Paxinos and Watson 2014).

#### Statistical analysis

To compare behavior after injections with the different doses of U50,488 across the two NAc shell subregions, data were analyzed using multiple two-way ANOVAs with dose as the within-subject measure, and cannnulation area as the between-subject measures. The dependent variables - total consumption, final response ratio and break point - were analyzed separately from each other.

## Results

### Experiment 1 – Effects of KOR stimulation on behavior in a light-dark box and ethanol drinking

#### Histology and ethanol drinking

**Fig. 3:**
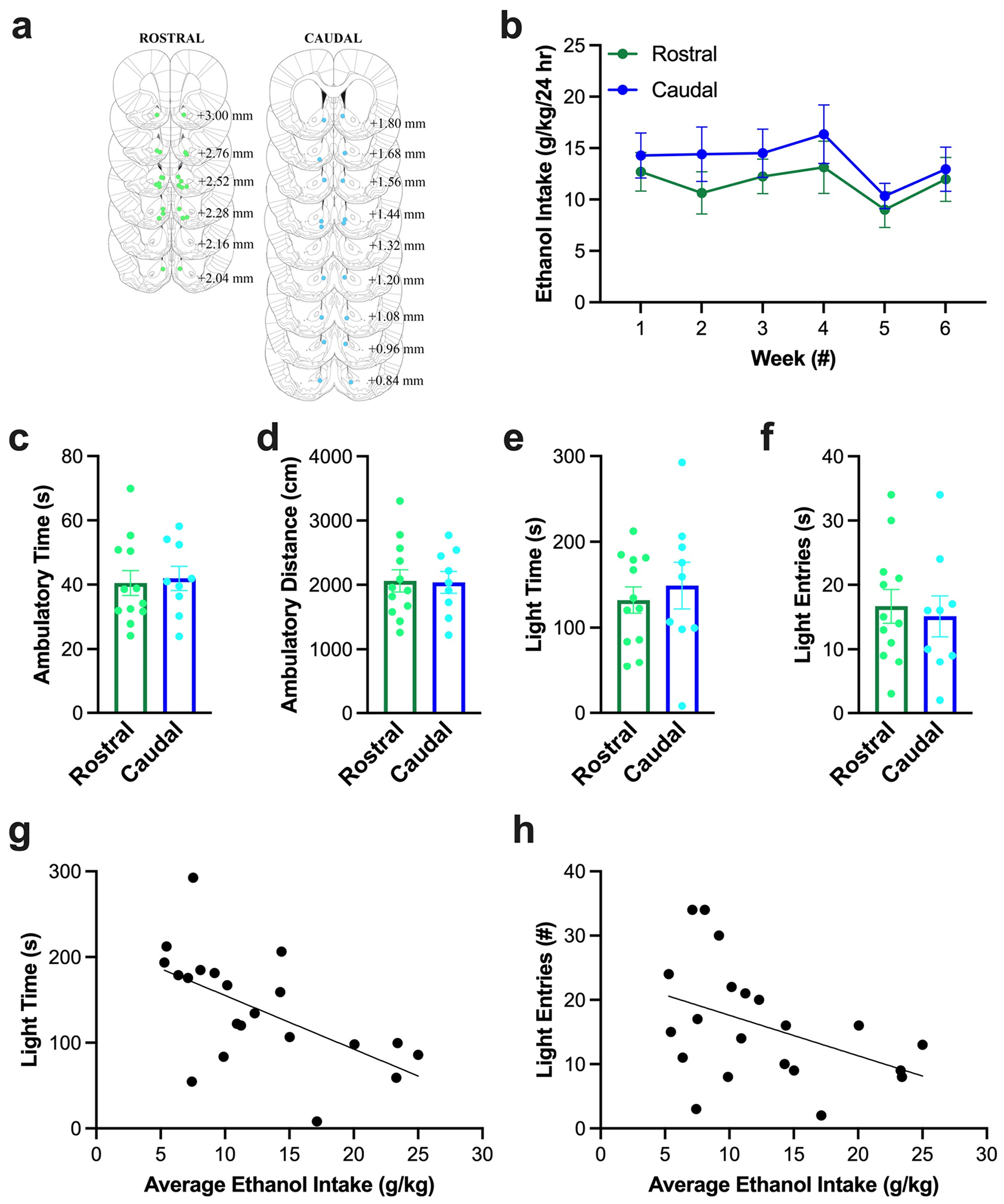
Behavior of rats in Experiment 1 prior to testing effects of U50,488. (**a**) Histological analysis confirmed that injections for subjects included in the analysis were made into medial nucleus accumbens (NAc) shell, with rostral injections being made between Bregma + 3.00 and + 2.04 mm, and caudal injections being made between Bregma + 1.80 – +0.84 mm. Dots indicate placement of on-target injections. Light green = rostral, light blue = caudal. (**b**) Ethanol drinking in the six weeks prior to microinjections was not significantly different between groups or across weeks. (**c**) Behavior in the lightdark box during the first session (“acclimation”) was not significantly different between rostral and caudal NAc shell groups for ambulatory time, (**d**) ambulatory distance, (**e**) time in the light chamber (“light time”), or (**f**) entries into the light chamber (“light entries”). (**g**) Average weekly ethanol intake prior to testing significantly predicted 34.2% of the variation in the time in the light chamber. (**h**) Average weekly ethanol intake prior to testing showed a trend for predicting 18% of the variation of entries into the light chamber. Values are Mean ± S.E.M

Histological examination confirmed that injections for subjects included in the analysis were made into medial NAc shell, with rostral injections being made between Bregma + 3.00 and + 2.04 mm, and caudal injections being made between Bregma + 1.80 – +0.84 mm (Fig. 3 (a)). Ethanol drinking in the six weeks prior to microinjections averaged 12.4 ± 1.3 g/kg/d and was not significantly different between groups (*F*_1, 19_ = 0.65, *p* = 0.431) or across weeks (*F*_1, 19_ = 2.82, *p* = 0.109) (Fig. 3 (b)), indicating that baseline drinking was stable and similar between subjects prior to being injected in the rostral or caudal NAc shell.

#### Acclimation to the light-dark box

As with ethanol drinking, behavior in the light-dark box during the first session (“acclimation”) was not significantly different between rostral and caudal groups for ambulatory time (*t*_19_ = −0.26, *p* = 0.795) (Fig. 3 (c)), ambulatory distance (*t*_19_ = 0.09, *p* = 0.928) (Fig. 3 (d)), time in the light chamber (*t*_19_ = −0.57, *p* = 0.573) (Fig. 3 (e)), or entries into the light chamber (*t*_19_ = 0.38, *p* = 0.708) (Fig. 3 (f)), indicating that baseline behavior in the light-dark box was similar between subjects prior to being injected in the rostral or caudal NAc shell. Examining the relationship of these behaviors with average weekly ethanol intake prior to testing, ethanol significantly predicted 34.2% of the variation in the time in the light chamber (*R*^2^ = 0.34, *F*_1, 19_ = 9.88, *p* = 0.005), such that higher ethanol drinking predicted less time in the light chamber (ß = −0.59, *p* = 0.005) (Fig. 3 (g)). Similarly, ethanol showed a trend for predicting 18% of the variation of entries into the light chamber (*R*^2^ = 0.18, *F*_1, 19_ = 4.18, *p* = 0.055), such that higher ethanol drinking predicted fewer entries into the light chamber (ß = −0.43, *p* = 0.055) (Fig. 3 (h)). On the other hand, ethanol did not significantly predict ambulatory time (*R*^2^ = 0.03, *F*_1, 19_ = 1.59, *p* = 0.223) or ambulatory distance (*R*^2^ = 0.03, *F*_1, 19_ = 1.51, *p* = 0.234).

#### Effects of KOR stimulation on behavior in a light-dark box

**Fig. 4:**
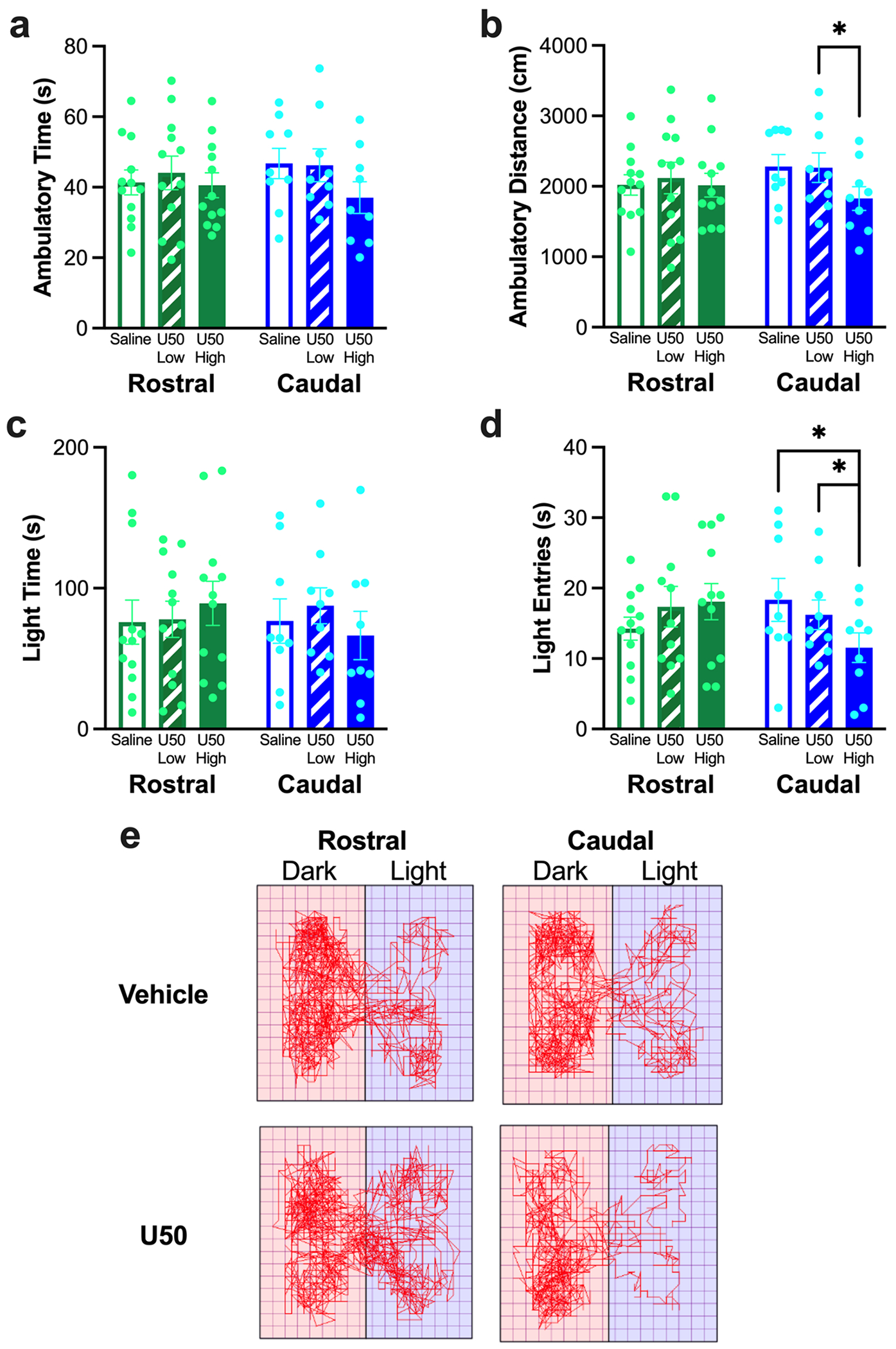
Behavior of rats in Experiment 1 in the light-dark box during the tests after injection of U50,488 (U50) in the rostral or caudal nucleus accumbens (NAc) shell. (**a**) Injection of U50,488 into neither the rostral nor the caudal NAc shell led to any significant difference in ambulatory time. (**b**) Injection of U50,488 into the caudal but not rostral NAc shell led to less distance traveled after the high dose of U50,488 compared to the low dose. (**c**) Injection of U50,488 into neither the rostral nor the caudal NAc shell led to any significant difference in time in the light chamber. (**d**) Injection of U50,488 into the caudal but not rostral NAc shell led to fewer entries into the light chamber after the high dose compared to both the low dose and saline vehicle. (**e**) Representative activity traces of two rats after injection with the high dose of U50,488 or saline vehicle in the rostral or caudal NAc shell. Pink (left) side = dark chamber, violet (right) side = light chamber. Values are Mean ± S.E.M. *p < 0.05

For behavior in the light-dark box during the tests after injection of U50,488 into the rostral NAc shell, there was no difference in ambulatory time (*F*_2, 13.95_ = 0.58, *p* = 0.498) (Fig. 4 (a)), ambulatory distance (*F*_2, 14.18_ = 0.25, *p* = 0.686) (Fig. 4 (b)), time in the light chamber (*F*_2, 22_ = 0.94, *p* = 0.404) (Fig. 4 (c)), or entries into the light chamber (*F*_2, 13.02_ = 0.75, *p* = 0.437) (Fig. 4 (d)), although the high dose did increase the number of entries into the light chamber by 27%. In contrast, after injection of U50,488 into the caudal NAc shell, there was a significant difference in ambulatory time (*F*_2, 16_ = 4.70, *p* = 0.025) (Fig. 4 (a)), although pairwise comparisons revealed no significant difference between any individual conditions. There was also a significant difference in ambulatory distance (*F*_2, 16_ = 6.36, *p* = 0.009), with less distance traveled after the high dose of U50,488 compared to the low dose of U50,488 (*p* = 0.031), and a trend for less distance traveled after the high dose of U50,488 compared to saline vehicle (*p* = 0.057) (Fig. 4 (b)). While there was no difference in time in the light chamber (*F*_2, 16_ = 1.42, *p* = 0.270) (Fig. 4 (c)), there was a significant difference in entries into the light chamber (*F*_2, 16_ = 7.06, *p* = 0.006), with fewer entries into the light chamber after the high dose of U50,488 compared to saline vehicle (*p* = 0.039) and the low dose of U50,488 (*p* = 0.045) (Fig. 4 (d)). These differences in behavior in the light-dark box during the tests after injection of U50,488 can be visualized through representative activity traces (Fig. 4 (e)).

#### Effects of KOR stimulation on ethanol drinking

**Fig. 5:**
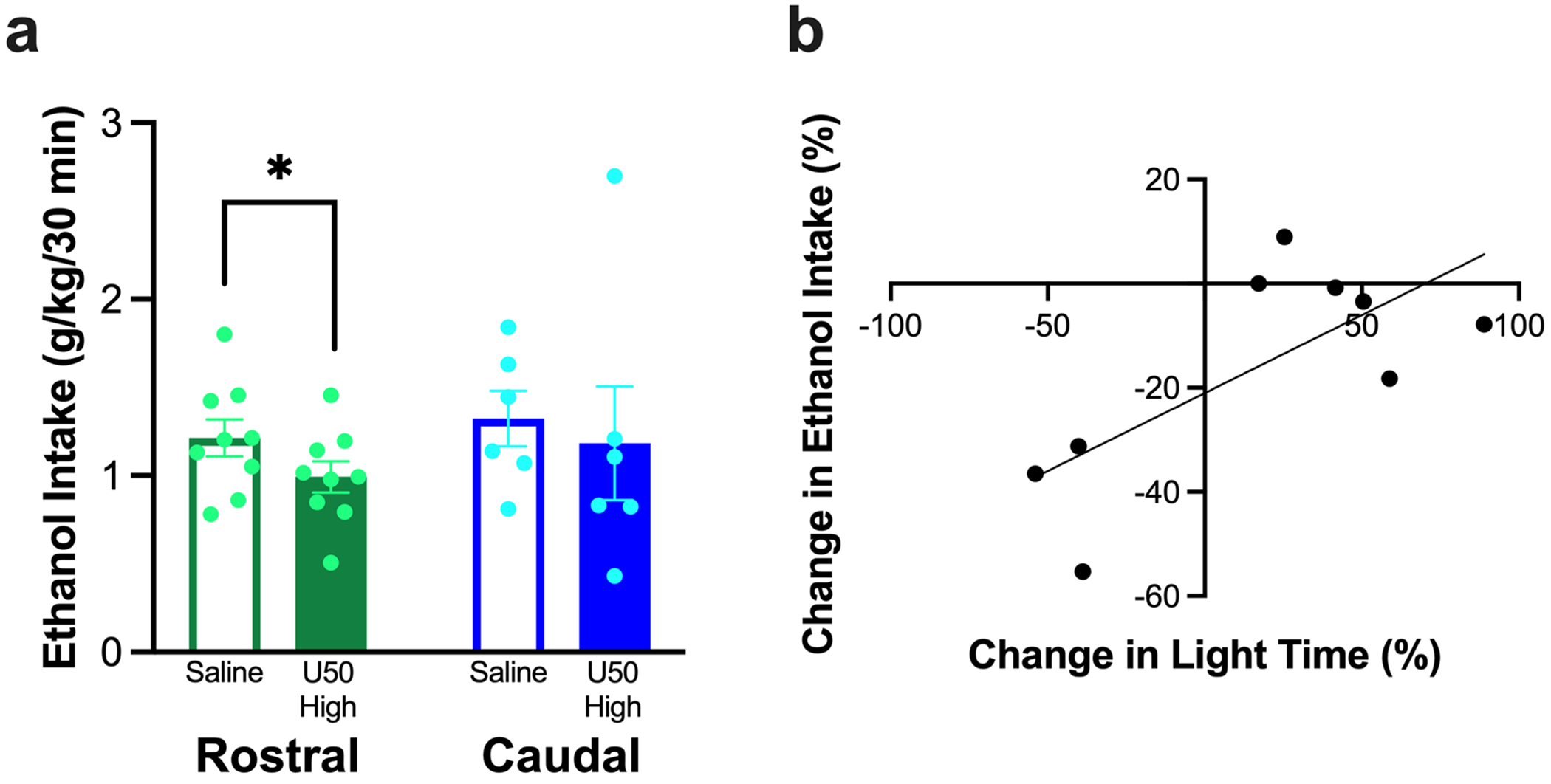
Behavior of rats in Experiment 1 in relation to ethanol drinking after injection of U50,488 (U50) in the rostral or caudal nucleus accumbens (NAc) shell. (**a**) Injection of U50,488 in the rostral but not caudal NAc shell significantly reduced ethanol drinking. (**b**) Time in the light chamber after injection of U50,488 in the rostral NAc shell significantly predicted 50.9% of the variation in the percent change in ethanol drinking. Values are Mean ± S.E.M. *p < 0.05

Whereas ethanol drinking after injection with U50,488 in the rostral NAc shell was significantly reduced (*t*_7_ = −2.07, *p* = 0.039), drinking after injection in the caudal NAc shell was not significantly different (*t*_5_ = −0.45, *p* = 0.336) (Fig. 5 (a)). Intake of simultaneously-available water and chow was not significantly changed after injection into the rostral NAc shell (water: *t*_7_ = 0.68, *p* = 0.521, chow: *t*_7_ = 1.58, *p* = 0.158) or the caudal NAc shell (water: *t*_5_ = −0.75, *p* = 0.486, chow: *t*_5_ = −2.25, *p* = 0.074) (data not shown). Notably, while the percent change in ethanol drinking after injection of U50,488 in the rostral NAc shell was not significantly predicted by the percent change in ambulatory time (*R*^2^ = 0.04, *F*_1, 7_ = 0.25, *p* = 0.632), ambulatory distance (*R*^2^ = 0.02, *F*_1, 19_ = 0.13, *p* = 0.730), or entries into the light chamber (*R*^2^ = 0.02, *F*_1, 19_ = 1.55, *p* = 0.253), 50.9% of the variation in the percent change in ethanol drinking was significantly predicted by time in the light chamber (*R*^2^ = 0.51, *F*_1, 7_ = 7.27, *p* = 0.031), such that a larger reduction in time in the light chamber predicted a larger reduction in ethanol drinking (ß = 0.71, *p* = 0.031) (Fig. 5 (b)). The percent change in ethanol drinking after injection of U50,488 in the caudal NAc shell was not significantly predicted by the percent change in ambulatory time (*R*^2^ = 0.05, *F*_1, 4_ = 0.22, *p* = 0.662), ambulatory distance (*R*^2^ = 0.04, *F*_1, 4_ = 0.18, *p* = 0.691), entries into the light chamber (*R*^2^ = 0.02, *F*_1, 4_ = 0.07, *p* = 0.800), or time in the light chamber (*R*^2^ = 0.03, *F*_1, 4_ = 0.14, *p* = 0.728) (data not shown).

### Experiment 2 – Effects of KOR stimulation on lever-pressing for sucrose self-administration

#### Cannula placement confirmation and sucrose drinking

**Fig. 6:**
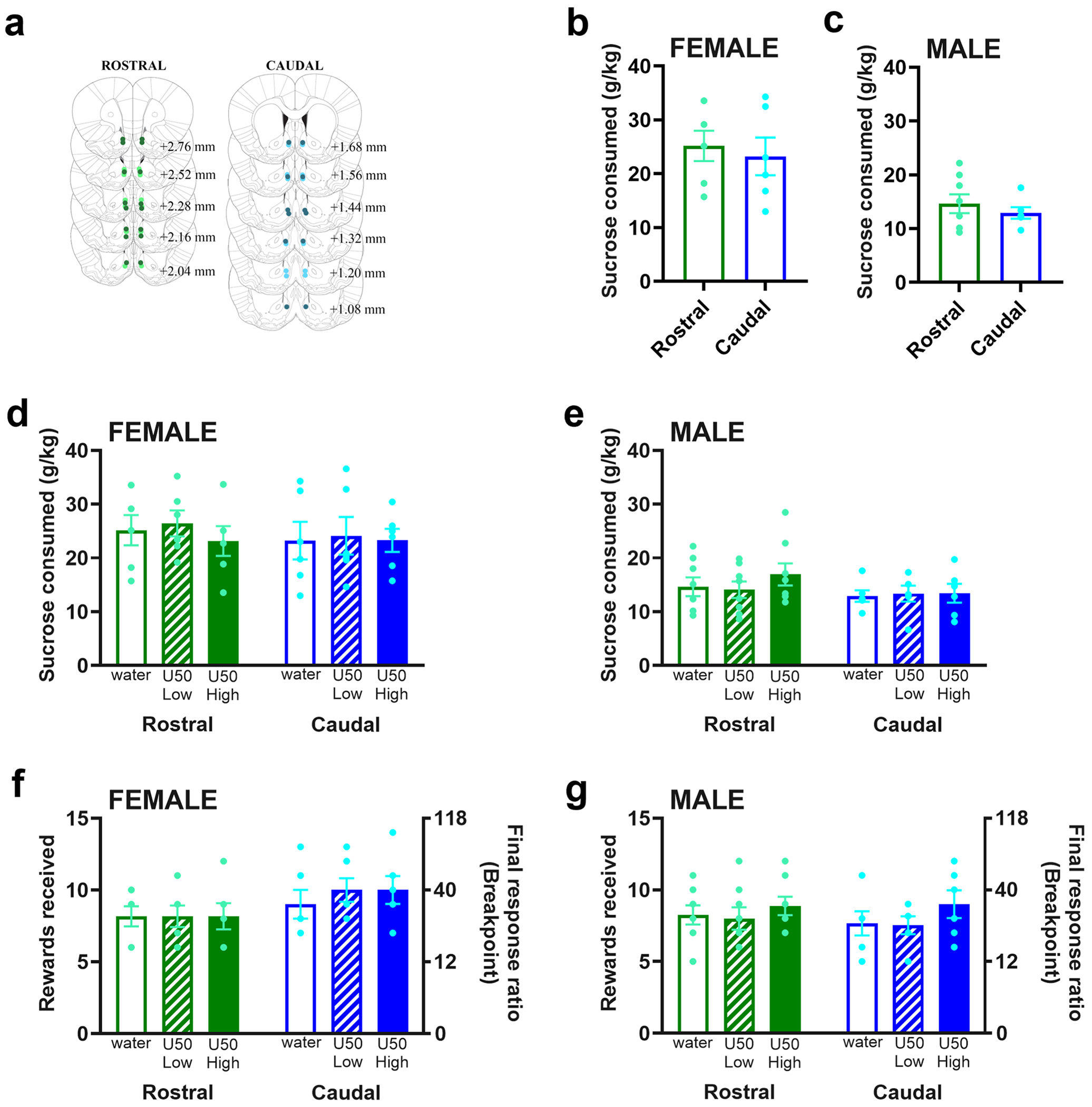
Behavior of rats in Experiment 2 in relation to sucrose consumption and motivation to consume sucrose. **(a)** Cannula verification confirmed that injections for subjects included in the analysis were made into medial nucleus accumbens (NAc) shell, with rostral injections being made between Bregma + 2.76 and + 2.04 mm, and caudal injections being made between Bregma + 1.68 – +1.08 mm. Dots indicate placement of on-target injections. Light green = female rostral, dark green = male rostral, light blue = female caudal, dark blue = male caudal. **(b)** There were no significant differences in sucrose consumption between rostral and caudal cannula placements in female or **(c)** male rats. **(d)** Sucrose consumption remained unaltered in female rats after low and high doses of U50,488 microinjected into the rostral and caudal NAc shell. **(e)** Similarly, male rats microinjected with U50,488 in rostral or caudal NAc shell did not exhibit any significant changes in sucrose consumption. **(f)** Final response ratio/rewards received was not significantly altered with U50,488 microinjection into the rostral or caudal NAc shell in female or **(g)** male rats

Examination of cannula placement confirmed that injections for subjects included in the analyses targeting the rostral NAc shell were made between Bregma + 3.00 and + 2.04 mm, and the caudal NAc shell were between Bregma + 1.80 – +0.84 mm (Fig. 6 (a)). Sucrose consumption between groups with rostral and caudal cannula placement was not significantly different [Female: (*t*_10_ = 0.43, *p* = 0.673), Fig. 6(b); Male: (*t*_12_ = 0.77, *p* = 0.458), Fig. 6(c)], indicating that baseline sucrose consumption was similar between subjects with cannulas in the rostral and caudal NAc shell.

#### Effects of KOR stimulation on sucrose consumption

In female rats, sucrose consumption was unaffected by injections in the rostral compared to caudal NAc shell [*F_1,10_* = 0.15, *p* = 0.707] or by U50,488 doses [*F_1.4,14.4_* = 0.72, *p* = 0.459] (Fig. 6(d)). Similarly, in male rats, injections in neither rostral nor caudal NAc shell altered sucrose consumption [*F_1,12_* = 0.92, *p* = 0.357] nor did U50,488 doses [*F_1.5,18.2_* = 1.09, *p* = 0.341] (Fig. 6(e)).

#### Effects of KOR stimulation on breakpoints/rewards received

Female rats after U50,488 injections in the rostral NAc shell exhibited comparable breakpoints and rewards received when compared with female rats after U50,488 injections in the caudal NAc shell [subregion: *F_1,10_* = 1.61, *p* = 0.233; dose: *F_1.9,19.5_* = 2.31, *p* = 0.127] (Fig. 6(f)). Similarly, male rats after U50,488 injections in the rostral compared to caudal NAc shell exhibited no differences in breakpoints and rewards received [subregion: *F_1,12_* = 0.129, *p* = 0.725; dose: *F_1.5,18_* = 2.52, *p* = 0.119] (Fig. 6(g)).

## Discussion

In the current study, we found significant differences in female rats in the effects of KOR stimulation in the rostral compared to caudal NAc shell on anxiety-like or approach-avoidance behavior in a light-dark box and ethanol drinking but not on measures of sucrose drinking. Specifically, injection of U50,488 into the caudal but not rostral NAc shell reduced the number of entries into the light chamber of a light-dark box and led to a trend for reduced distance traveled in the light-dark box. In contrast, injection of U50,488 into the rostral but not caudal NAc shell significantly reduced ethanol drinking. Moreover, ethanol consumption was inversely related to time in the light chamber during acclimation to the light-dark box, and the degree of response to U50,488 injection in the rostral NAc shell on ethanol drinking was predicted by the degree of response to U50,488 injection on time in the light chamber in the lightdark box.

We have previously reported that U50,488 injection in the caudal NAc shell of ethanol-naïve male rats led to reduced time spent in the light chamber of a light-dark box (Pirino et al. 2020). In the present study, the effect in female rats with a history of ethanol drinking was similar, with U50,488 injection resulting in a reduction in the number of entries into the light chamber. Interestingly, while the time spent in the light chamber after saline vehicle was roughly similar between the past males (Pirino et al. 2020) and current females (~ 80–100 s), the number of entries into the light chamber was somewhat higher in the past males (~ 25; (Pirino et al. 2020) compared to the current females (~ 15–20). This may have contributed to the difference in effect of KOR stimulation and could be accounted for by the trend for reduced entries into the light chamber that we observed after higher ethanol drinking. Additionally, with females generally being less sensitive than males to the behavioral effects of U50,488 (Conway et al. 2019; Jacobson et al. 2020b), the ability of ethanol exposure to upregulate KOR levels in the NAc (Cuitavi et al. 2024; Niinep et al. 2021; Pirino et al. 2024) may have also contributed to the sex-related effect of U50,488 on the exact measure changed in the light-dark box. While we did not find that injection of U50,488 in the rostral NAc shell led to anxiolytic or approach behavior, it should be noted that this effect in males was previously identified through testing in an open field rather than a light-dark box (Pirino et al. 2020) and that the U50,488 injection in the rostral NAc shell in the present study did increase the number of entries into the light chamber by 27%. Our current finding that U50,488 in the caudal NAc shell somewhat reduced distance traveled in the light-dark box also parallels the prior finding in males that U50,488 in the caudal NAc shell reduced distance traveled in an open field (Pirino et al. 2020). Given this similarity in the behavioral changes between females and males, this suggests that U50,488 in the rostral NAc shell of female rats might lead to anxiolytic behavior in tests beyond the lightdark box, although this remains to be determined.

The finding that injection of U50,488 into the rostral but not caudal NAc shell significantly reduced ethanol drinking is consistent with our prior findings in low-drinking female rats (Pirino et al. 2024). We have previously observed across multiple cohorts of Long-Evans rats drinking ethanol under an intermittent-access procedure that females cluster into low drinkers, which drink around 7g/kg/day, and high drinkers, which drink around 16 g/kg/day (Pirino et al. 2022, 2024). We also found that these drinkers exhibit different ethanol drinking responses to injection of U50,488 in the rostral NAc shell, with low drinkers showing a reduction in ethanol drinking (Pirino et al. 2024). While the drinking levels of our current cohort of females could originally be parsed (again, by cluster analysis) into low drinkers and high drinkers, it was primarily the high drinkers that, for unrelated reasons, were ultimately removed from the analysis. This led to the remaining group being largely composed of low-drinking females. It is possible that results for effects of U50,488 on behavior in a light-dark box in females with a history of higher-level drinking could be different from what we report here. Nevertheless, the results confirm that effects of U50,488 in lower drinkers are consistent across cohorts and experiments. Moreover, they suggest that a change in ethanol drinking after KOR stimulation may be due in part to a change in anxiety-like behavior, as the degree of response to U50,488 injection in the rostral NAc shell on ethanol drinking was predicted by the degree of response to U50,488 injection on time in the light chamber in the light-dark box in the same animals. That is, many of the rats injected with U50,488 into the rostral NAc shell responded with an increase in time spent in the light chamber and also a small decrease in ethanol drinking, but a few rats responded with a reduction in time spent in the light chamber and also a large decrease in ethanol drinking, suggesting that rats that respond to U50,488 injection with more anxiety-like behavior also respond with less ethanol drinking. This would be consistent with numerous other studies modeling addiction vulnerability that report associations between anxiety-like behaviors and ethanol intake in male mice and rats (Anderson et al. 2016; Chappell et al. 2013; Karkhanis et al. 2016; Rose et al. 2016) and female rats (Izidio and Ramos 2007).

The finding that neither sucrose consumption nor motivation to self-administrer sucrose was altered by activation of the KOR in the rostral or caudal NAc shell is consistent with our prior finding that injection of U50,488 in neither the rostral nor the caudal NAc shell affected drinking of intermittent access sucrose in the home cage (Pirino et al. 2024). These data together suggest that although KOR activation in the NAc shell can regulate negative affect and hedonic behaviors, the influence is selective towards anxiety-like behaviors, place or taste preference behaviors, and ethanol consumption. Activation of the KOR, at least in the NAc shell, does not seem to affect intake or self-administration of a natural reward. Indeed, the KOR system is known to be stress-responsive and therefore the regulatory control of KOR may be more highly activated following stress exposure, including exposure to stress-inducing environments, such as the light-dark box, and repeated withdrawal cycles from ethanol. Indeed, KOR inhibition in non-dependent animals or animals that are stress-naive has not consistently been found to alter ethanol consumption or anxiety-like behavior (Karkhanis et al. 2016; Rose et al. 2016; Walker et al. 2011). To this end, many studies have highlighted the role of KOR in promoting hyperkatifeia and negative reinforcement [*see* (Koob 2021) *for review*].

Overall, our results confirm and extend previous findings regarding the effects of KOR activation in subregions of the NAc shell on affective and motivated behavior. Activation of the KOR in the caudal NAc shell of ethanol-drinking female rats promoted negative affect as demonstrated via an enhancement in anxiety-like behavior in a light-dark box. Moreover, these changes in affective behavior after activation of the KOR may influence changes in ethanol drinking. In light of interest in stress-related neuropeptides as targets for the treatment of alcohol use disorder (Chavkin 2018; Heilig 2023), the current results support a continued focus on the KOR as a possible pharmacotherapeutic target.

## Data Availability

Raw data will be provided upon request.
